# Inhibition of WHSC1 Allows for Reprogramming of the Immune Compartment in Prostate Cancer

**DOI:** 10.3390/ijms22168742

**Published:** 2021-08-14

**Authors:** Muzamil Y. Want, Ellen Karasik, Bryan Gillard, A. J. Robert McGray, Sebastiano Battaglia

**Affiliations:** 1 Department of Immunology, Division of Translational Immuno Oncology, Roswell Park Comprehensive Cancer Center, Buffalo, NY 14263, USA; muzamil.want@roswellpark.org (M.Y.W.); AJRobert.McGray@RoswellPark.org (A.J.R.M.); 2 Department of Pharmacology and Therapeutics, Roswell Park Comprehensive Cancer Center, Buffalo, NY 14263, USA; Ellen.Karasik@RoswellPark.org (E.K.); Bryan.Gillard@RoswellPark.org (B.G.); 3 Department of Cancer Genetics and Genomics, Roswell Park Comprehensive Cancer Center, Buffalo, NY 14263, USA

**Keywords:** tumor microenvironment, prostate cancer, immunotherapy

## Abstract

Immunotherapy initially demonstrated promising results in prostate cancer (PCa), but the modest or negative results of many recent trials highlight the need to overcome the poor immunogenicity of this cancer. The design of effective therapies for PCa is challenged by the limited understanding of the interface between PCa cells and the immune system in mediating therapeutic resistance. Prompted by our recent observations that elevated WHSC1, a histone methyltransferase known to promote progression of numerous cancers, can silence antigen processing and presentation in PCa, we performed a single-cell analysis of the intratumoral immune dynamics following in vivo pharmacological inhibition of WHSC1 in mice grafted with TRAMP C2 cells. We observed an increase in cytotoxic T and NK cells accumulation and effector function, accompanied by a parallel remodeling of the myeloid compartment, as well as abundant shifts in key ligand–receptor signaling pathways highlighting changes in cell-to-cell communication driven by WHSC1 inhibition. This comprehensive profiling of both immune and molecular changes during the course of WHSC1 blockade deepens our fundamental understanding of how anti-tumor immune responses develop and can be enhanced therapeutically for PCa.

## 1. Introduction

Modulating the immune landscape of prostate cancer (PCa) to promote anti-tumor immunity has gained enthusiasm following the initial clinical success of trials testing Provenge and Prostvac VF to stimulate immune responses against prostate acid phosphatase (PAP) and prostate specific antigen (PSA), respectively. While several studies have reported favorable clinical outcomes, especially in patients with therapy-induced immune infiltration, PCa tumors typically show only low-level immune cell infiltration in the tumor microenvironment (TME).

WHSC1 is a histone methyltransferase that targets histone 3 lysine 36 (H3K36) and, to a lesser extent, histone 4 lysine 20 (H4K20). Elevated WHSC1 expression correlates with worse prognosis in a number of cancers [1,2,3,4,5,6,7] due to its oncogenic role in promoting cell growth and metastases; however, the magnitude of the effect and mechanism(s) of action of WHSC1 remain poorly understood. In studies designed to understand the interplay between PCa cells and the immune system, we recently demonstrated that elevated levels of the WHSC1 enzyme limit lymphocyte infiltration in PCa tumors, reduce antigen processing and presentation, as well as repress local activation of immune pathways [8].

We previously reported that WHSC1 inhibition increases the frequency of intratumoral CD8^+^ T cells [8]; however, the changes associated with the overall immune composition of the TME following WHSC1 blockade remain unknown. Here, we broadly investigate the immunological changes in the TME in vivo following pharmacological inhibition of WHSC1and report a phenotypic shift in infiltrating immune cells that reflects both increased cytotoxic T cell activity and local modulation of diverse myeloid cell subsets present within the tumor. Lastly, using pathway analysis of changes resulting from WHSC1 inhibition, we propose a signaling circuitry that defines immune activation and favors a potent anti-tumor response.

## 2. Results

### 2.1. Pharmacological Inhibition of WHSC1 Increases Survival and Immune Function In Vivo

**Figure 1:**
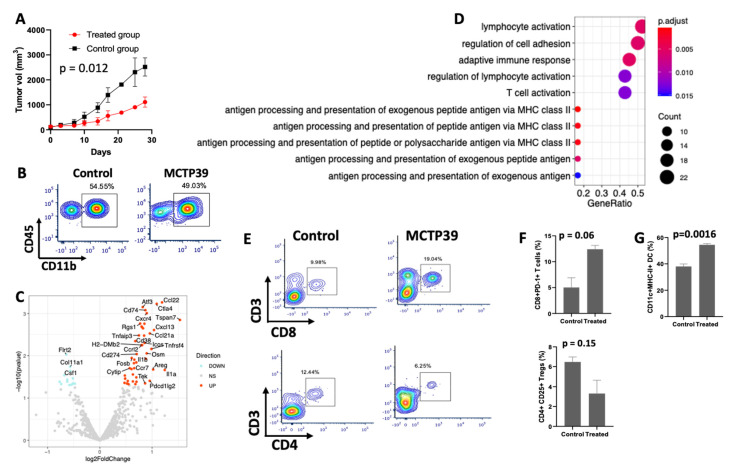
Effect of WHSC1 inhibition in vivo. (**A**) Tumor growth in MCTP39-treated mice. Tumor growth was measured over time in mice grafted subcutaneously with TRAMP C2 cells. Black and red lines indicate control and MCTP39-treated mice, respectively. Each time point reports the average tumor growth in at least two mice. (**B**) Intratumoral CD45^+^CD11b^+^ cell abundance in tumors from control and mice treated with MCTP39. Percentage of gated cells over total number of cells is shown. (**C**) Volcano plot showing the results from differential gene expression analysis of the Nanostring data. Red and cyan dots indicate up- and downregulated genes, respectively. Note that not all gene names are shown to reduce visual crowding. (**D**) Functional enrichment analysis of the differentially expressed genes from Nanostring data shown in **C** using murine gene ontology signatures. (**E**) Flow cytometry analysis of CD3^+^CD8^+^ and CD3^+^CD4^+^ T cells in control (left) and treated (right) samples. (**F**) Quantification of PD-1+ CD8^+^ T cells (top) and CD4^+^CD25^+^Tregs (bottom). (**G**) Quantification of CD11c^+^MHC-II^+^ dendritic cells. Each bar represents the mean and SD of at least two biological replicates. *p* values were calculated with Students’ *t* test.

Immunocompetent C57B/6 mice were grafted with TRAMP C2 cells [9] and treated with MCTP39 for 4 weeks. MCTP39 treatment resulted in tumor growth suppression, with an average reduction in tumor size of 66% in the treated group compared to the control (Figure 1A). While our previous findings demonstrated an increase in intratumoral lymphocytes in response to MCTP39 treatment [8], CD11b^+^ myeloid cells comprised the majority (~60%) of infiltrating immune cells in TRAMP C2 tumors regardless of treatment (Figure 1B). These results are consistent with previous reports describing high levels of monocytes/macrophages in the TME of prostate tumors [10,11] and melanoma [12]. To investigate whether MCTP39 treatment was impacting the myeloid cells on a global level in growing tumors, bulk RNA from resected tumors was processed and analyzed using the Nanostring immune myeloid panel gene set. Transcriptional changes following MCTP39 treatment showed increased expression of MHC genes *(H2-DMb2*, *H2-Aa,* and *H2-Ab1*) (Figure 1C, Supplementary File S1), consistent with our previous results [8], in addition to upregulation of Cd74, the invariant chain of MHC II [13] (Figure 1C). Chemokines involved in the recruitment of multiple immune cell subsets (Cxcr4, Cxcl13, Ccl22) were upregulated in parallel with the co-stimulatory molecules Icos and the checkpoint receptor Ctla4, suggesting complex immunological changes in the tumor microenvironment (TME) in response to treatment. (Figure 1C). Other upregulated genes were related to myeloid cell lineage commitment (*Irf8*) [14], myeloid/macrophage function (*Il1a*, *Il1b*, *Osm*), cellular mobility (*Tspan7*), as well as Cd38, which can be expressed by macrophages and correlates with improved outcome as observed in liver cancer [15], but can also function to breakdown ADP and suppress T cells’ function [16] (Figure 1C). Interestingly, Pd-l1 (Cd274) was also upregulated (Figure 1C), consistent with the induction of adaptive immune resistance mechanisms following local immune activation [17] and anti-tumor immunity (Figure 1C). Functional enrichment analysis, adjusted for the size of the Nanostring gene set, revealed that the top enriched pathways were associated with the activation of adaptive and innate immune responses in parallel with increased antigen processing and presentation (Figure 1D). Overall, these results point towards a parallel reshaping of the lymphoid and myeloid compartments following pharmacological inhibition of WHSC1 in vivo.

### 2.2. WHSC1 Inhibition Alters the Infiltration of T Cells and Modulates DC Function

To further investigate immunological changes resulting from WHSC1 inhibition, CD45+ cells were isolated from tumors after 4 weeks of treatment with MCTP39 and analyzed via flow cytometry. While the overall number of CD45^+^CD3^+^ lymphocytes was constant across conditions (Figure S1A), MCTP39 treatment led to a decrease in total CD4^+^ TILs, accompanied by a parallel increase in CD8^+^ TILs (Figure 1E). Within CD8^+^ T cells, we identified an increase in the frequency of PD-1^+^ CD8^+^ TIL following MCTP39 treatment (Figure 1F, upper panel). Additionally, within the CD4^+^ T cell compartment, we observed a decrease in CD4^+^CD25^+^ Tregs in the treatment group (Figure 1F, lower panel), although this was not statistically different. Lastly, we observed a significant increase in MHC-II on CD45^+^CD11c^+^ DCs in treated mice compared to controls. (Figure 1G), consistent with increased localization of immunostimulatory DCs in the TME following treatment. The elevation in MHC-II and the increased CD8^+^ T cell infiltration are supported by the gene expression data from Nanostring (Figure 1C,D) and suggest an active reshaping of the immune compartment from immuno-suppressive to immuno-permissive, thus enhancing the anti-tumor functional activity of infiltrating immune cells.

### 2.3. Heterogeneous Myeloid Populations Infiltrate Prostate Tumors

**Figure 2:**
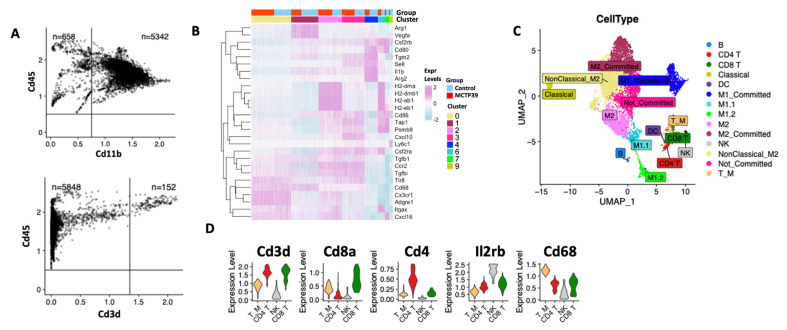
scRNASeq analysis of tumor-infiltrating immune cells. (**A**) Exploratory scatter plot showing the proportions of CD45^+^CD11b^+^ myeloid cells (top) and CD45^+^CD3^+^ lymphocytes (bottom) in the samples used for scRNASeq. (**B**) Expression pattens for the panel of genes used to classify myeloid cells. Purple and light blue cells indicate high and low expression, respectively. The annotation on top indicates the cluster assignments and the group (control, MCTP39). (**C**) UMAP representation of the final annotation for all the cell types identified. (**D**) Violin plots showing the expression of markers used to classify CD8^+^, CD4^+^, NK, and T_M cells.

Since treatment of tumor-bearing mice with MCTP39 affects intratumoral immune pathways globally [8] and alters multiple infiltrating immune cell subsets, we sought to investigate how the broader immune landscape of tumors was being impacted by MCTP39. To this end, we interrogated intratumoral CD45^+^ single cells from control and MCTP39-treated mice using scRNASeq. Following QC, preprocessing, and removal of dead and low-quality cells, 3000 cells per condition were used for downstream analyses. Consistent with the results from flow cytometry, the majority of the CD45^+^ population comprised CD11b^+^ myeloid cells, with limited infiltration by CD3^+^ T lymphocytes (Figure 2A), and subsequent cluster analysis separated the lymphoid from the myeloid compartments (Figure S2).

In order to gain a more granular understanding of the immune populations infiltrating PCa tumors, the lymphocytic and myeloid compartments were further annotated. The myeloid populations span across nine closely aggregated clusters, with two clusters, 4 and 9, spatially separated from the others (Figure 2B, Figure S2). The presence of closely related myeloid cells is consistent with the biology of myeloid cells, which exist on a spectrum of differentiation that, depending on the stimulus, can commit to different functional programs, including diverse DC lineages, or to M1/M2 polarization. We therefore utilized a panel of established markers (Supplementary File S2) and their corresponding gene expression patterns to annotate the identified populations to the correct clusters.

Given the overall abundance of myeloid cells in PCa tumors, myeloid cells were reclassified as shown in Figure 2B. Cluster 0 cells were positive for Cd68, Cd49/Adgre1, Cx3cr1, Cd11c/Itgax, and Cxcl16 and negative for Ccr2 and Cd62l/Sell, suggesting that these cells were non-classical monocytes committed to an M2 phenotype. Cluster 1 contained cells that expressed high levels of Cd68, VEGFa, and Arg1, in parallel with low levels of Cd11c/Itgax and Cxcl16, pointing towards partially polarized M2 macrophages. Cluster 2 was positive for MHC class II, Cx3cr1, Cd49/Adgre1, Ccl5, and Cxcl16, representing M2 macrophages. Cluster 3 appeared to be a heterogeneous population with markers consistent with monocytes and macrophages, potentially indicating a non-committed population. Cells in cluster 4 were positive for Arg2, Il1b, Cd80, and Csf2rb, while still expressing Sell and were MHC II-negative, suggesting that they might be partially polarized M1 macrophages, as M1 macrophages can have low MHC expression, and were described as TAMs elsewhere [18]. Cluster 6 was positive for Csf2rb and Cd80, with high levels of MHC molecules, Cd11c/Itgax, Cd86, and detectable Il1b, suggesting almost fully polarized M1 macrophages. Cluster 7 contained cells positive for Cd80, Csf2rb, Cd86, and the chemokines Ccl5 and Cxcl16, with detectable MHC expression, suggesting a separate subset of polarized M1 macrophages. Cluster 8 had high levels of Cx3cr1, Cd49/Adgre1, and Tlr8, were negative for Cd86, and showed low levels of Cd68 and Cxcl16, suggesting non-classical monocytes committed to becoming M2 macrophages. Lastly, cluster 9 was positive for Ly6c1, with weak Csf2rb, Tgm2, and Cd86 expression, suggesting uncommitted classical monocytes.

Next, we profiled the lymphoid compartment. First, we classified the lymphocytic populations in clusters 5 and 10, successfully separating CD4^+^ from CD8^+^ T cells and NK cells (Figure 2C). Interestingly, cluster 5 also included a rare population of macrophage with modest Cd3 expression, which we classified as T cell-like macrophage (T_M), which have been previously described as having a cytotoxic and pro-inflammatory role [19,20]. In our samples, this population expressed Tnf, the degranulation marker Lamp1/Cd107a, the chemokine CcrR5, and other M1 markers such as Cd68 and IL1B (Figure 2D).

### 2.4. WHSC1 Inhibition Promotes Cytotoxic Functions

**Figure 3:**
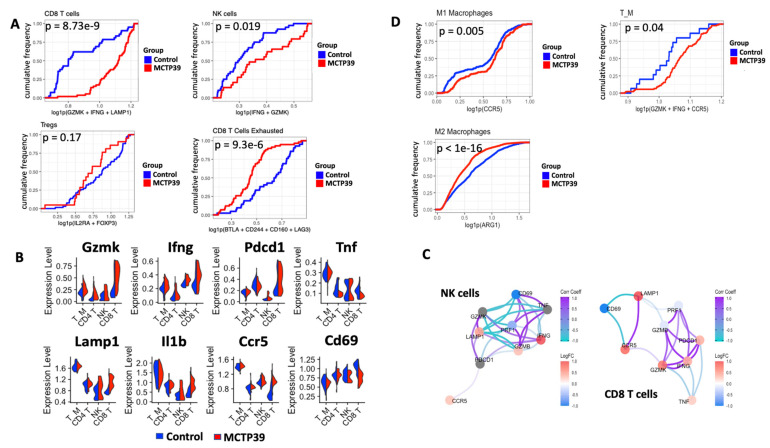
Cell population changes following WHSC1 inhibition. (**A**) Cumulative frequency of lymphocytic populations in control mice (blue) and MCTP39-treated mice (red). Population frequency (0 to 1) is shown on the y axis, expression levels of the markers in the axis name is shown on the x axis. Significance was calculated using the Kolmogorov–Smirnov test, with p values shown in the figure. (**B**) Expression levels of the markers used to classify activated lymphocytes. Each violin plot is split for the control group (blue) and the MCTP39-treated group (red). (**C**) Correlation network analysis of the cytotoxic markers expressed in NK (left) and CD8^+^ T cells (right). (**D**) Cumulative frequency of myeloid populations in the control (blue) and MCTP39-treated samples (red). Significance was calculated using the Kolmogorov–Smirnov test, with *p* values shown in the figure.

To further understand the antitumor activity of the identified immune cell subsets following MCTP39 treatment, we next evaluated the expression of cytotoxic markers in treated and control tumors. There was an increase in the frequency of both CD8^+^ T cells and NK cells expressing cytotoxic markers including granzymes, Ifn-γ, and Lamp 1 (Figure 3A,B). As MCTP39 alone does not affect the proliferation of CD8^+^ T or the levels of IFN-γ and TNF-α production when T cells are simulated in vitro (Figure S1B), this suggests that the observed activation of CD8^+^ T cells in vivo was not due to the direct activity of MCTP39. In parallel, we observed a modest reduction in the frequency of Il2ra^+^Foxp3^+^ Treg following MCTP39 treatment (Figure 3A), consistent with the initial flow cytometry results. Interestingly, this was accompanied by a significant decrease in the frequency of CD8^+^ T cells expressing the exhaustion markers Cd244, Btla/Cd272, and Cd160 (Figure 3A), suggesting reinvigoration and/or prolonged functionality of CD8^+^ T cells following treatment with MCTP39. CD4^+^ T cells and T_M cells also showed an increase in the degranulation marker Cd107a/Lamp1, Gzmk, and Ccr5 (Figure 3B), supporting the establishment of a pro-inflammatory TME following MCTP39 treatment. The complementary cytotoxic functions of CD8^+^ T cells and NK cells were investigated further by evaluating the coexpression of cytotoxic makers in these two cell types. NK cells showed a positive coexpression pattern for Cd69, Tnf, Ifng, Gmzb, and Prf1, with a separate pattern that included Gzmk, Lamp1, and Pdcd1 (PD-1) (Figure 3B,C). CD8^+^ T cells had a core pattern represented by Pdcd1, Gzmk, Gzmb, Ifn-γ, Prf1, with Lamp1 and Ccr5 representing a separate coexpression cluster (Figure 3B,C), suggesting that NK and CD8^+^ T cells possessed complementary, yet partially diverse, cytotoxic profiles. Overall, these results suggest that pharmacological inhibition of WHSC1 reshaped the lymphoid compartment of PCa towards a more immunopermissive and cytotoxic phenotype.

### 2.5. Inhibition of WHC1 Functionally Reprograms the Myeloid and Lymphoid Compartments

To better understand the changes in the myeloid compartment following treatment with MCTP39, we evaluated the frequency of activated M1 macrophages expressing Ccr5 and the frequency of immunosuppressive M2 macrophages positive for Arg1. In line with improved T cell activity and local inflammation, MCTP39 treatment led to a higher frequency of pro-inflammatory M1 cells and a corresponding decrease in Arg1-expressing M2 macrophages (Figure 3D). Interestingly, the T_M population expressing pro-inflammatory markers such as Gzmk, Ifng, and Ccr5 was increased in the treated group (Figure 3D), suggesting a global reprogramming of the myeloid compartment that favored a pro-inflammatory phenotype.

**Figure 4:**
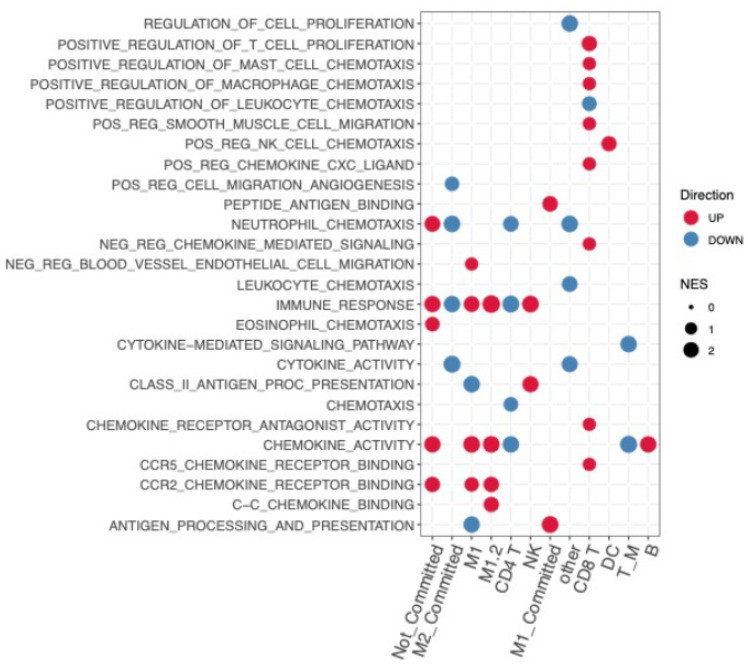
Gene set variation analysis. Results from GSVA analysis using the top up- and downregulated genes for each cell type/cluster. Red (positive) and blue (negative) dots indicate significant pathway normalized enrichment scores (NES), obtained by comparing the control to MCTP39-treated samples. Each cell type (x axis) was analyzed individually.

To further profile the transcriptional changes occurring after WHSC1 inhibition and the accompanying impact on intratumoral immunological functions, we performed differential gene expression analysis followed by functional enrichment analysis using the cell types identified above (Figure 4). In order to add valuable biological interpretation to the effect of MCTP39 in vivo, we used GSVA analysis to score pathway activity and the corresponding changes in each cell type. Following WHSC1 inhibition, clusters containing M1 and M2 macrophages had elevated expression of genes associated with chemokine activity and immune response. Conversely, M2_committed cells demonstrated an overall reduction in cytokine signaling, antigen presentation, and chemotaxis. Not committed cells appeared to increase the expression of genes associated with immune pathways, and DCs increased their ability to promote NK cells chemotaxis. CD8^+^ T cells revealed a strong upregulation of chemotaxis, migration, and immune response, in contrast to CD4^+^ T cells which showed a downregulation of genes involved in chemotaxis and immune response (Figure 4). Overall, these results suggest that inhibition of WHSC1 in vivo not only impacted lymphocyte function, but also led to an efficient reprogramming of the myeloid compartment, increasing antigen processing and presentation and limiting immunosuppressive transcriptional programs from M2 macrophages.

### 2.6. Ligand–Receptor Networks Reveal Rewiring of the Immune Circuitry in Response to Inhibition of WHSC1

**Figure 5:**
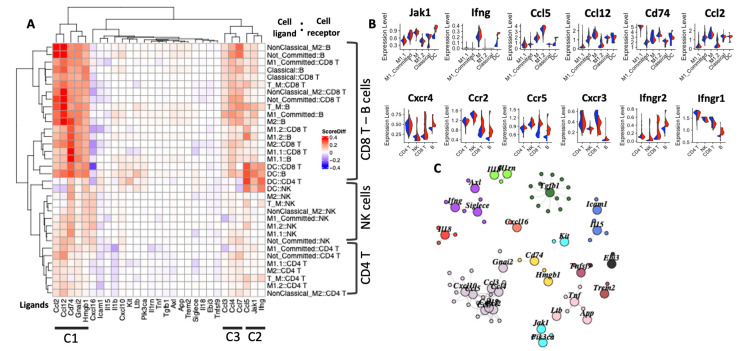
Ligand–receptor analysis. (**A**) Heatmap representing the difference in score between the control and MCTP39-treated samples. Red and blue cells denote that scores are higher or lower, respectively, following MCTP39 treatment and indicate a potential increase in cell-to-cell communication via a specific ligand–receptor interaction. Ligands are assumed to be produced by myeloid cells (see previous results), and the top changing ligands are listed at the bottom. The row names indicate the cell pair considered. The most represented receptor-expressing cell types are highlighted on the right side. (**B**) Violin plots showing the expression levels of the ligand and receptors for the top results shown in the heatmap. Cell types are shown on the x axis. (**C**) Cluster analysis of the ligand–receptor groups representing an overview of the multiple ligand –receptor interactions possible. Colors indicate different clusters.

While functional enrichment analysis revealed the upregulation of immune pathways following MCTP39 treatment, cell signaling events brought about through ligand–receptor interactions ultimately determine cellular response. In the attempt to profile the changes governing anti-tumor immune responses in the treated group, we compiled a list of ligand–receptor pairs [21] for chemokines and cytokine expressed within the myeloid and lymphoid compartments. We then scored each ligand–receptor interaction and compared the scores between treated and untreated samples to identify potential changes in pro-inflammatory signals elicited by MCTP39. The results separated cellular stimuli for CD8^+^ T cells, NK, and CD4^+^ T cells and defined three main clusters of signaling driven by Ccl2, Ccl12, Cd74, Gnai2, and Hmgp1 (C1), by Ifn-γ, Jak1, and Ccl5 (C2), and by Ccl3, Ccl4, and Ccl7 (C3). As shown in Figure 5A, signaling from C1 appeared to target mostly CD8^+^ T cells and B cells, independently of the cell type producing the ligand. Conversely, signaling from C2 appeared to originate from DCs and targeted multiple cell types (Figure 5A). Similar to C1, C3 broadly targets lymphocyte subsets through interactions with diverse myeloid cell subtypes. When key receptors involved in these signaling pathways were compared using gene expression, the levels of Cxcr4 and Ccr2 (C1) were elevated in CD8+ T cells and B cells, which also showed increased levels of Ifn-γ receptor and Ccr5 (Figure 5B). Similarly, Jak1, Ifn-γ, and Ccl5 expression levels (C2) were elevated in multiple myeloid cell subsets following MCTP39 treatment. In contrast, genes associated with cluster C3 did not show remarkable changes across individual myeloid cell subsets following MCTP39 treatment, suggesting that changes associated with this cluster may be the result of the collective signaling events occurring through multiple cellular interactions. To this end, since a single ligand can bind to multiple receptors, and vice versa, we performed a network analysis with clustering of the ligand–receptor pairs mentioned above and identified 12 clusters by which signals were propagated, with the largest group containing Cxcl/Ccl cytokines (Figure 5C). Overall, these results suggest that changes in the immune circuitry following WHSC1 inhibition in vivo were driven by a rewiring of the signaling from macrophage subsets and DCs, to ultimately elicit anti-tumor effects from T, NK, and B cells.

## 3. Discussion

We recently demonstrated that WHSC1 inhibition potently increases antigen processing and presentation via an elegant epigenetic remodeling of prostate cancer cells that increases MHC expression and antigen presentation, accompanied by an increase in tumor-infiltrating CD8^+^ T cells [8]. Interestingly, we also observed that when tumors were grafted in immunodeficient NSG mice, no tumor growth inhibition was observed [8], suggesting that at least part of the anti-tumor effect of MCTP39 was mediated through the activity of a functional immune system. While recognizing that both mouse models were grafted subcutaneously (SQ), it is well established that SQ tumors are still subject to the immune-driven anti-tumor effect in numerous cancer indications [8,12,22,23,24,25,26,27] and include a rich and heterogeneous immune compartment reshaped by therapeutic interventions. For these studies, we used the syngeneic prostate cancer cell line TRAMP C2 [9] that can be grafted in immunocompetent mice, thus permitting tumor-infiltrating immune cells to be broadly profiled in response to therapy.

Previous studies employing single-cell analyses identified large myeloid and lymphoid infiltrates in the prostate tumors of 13 patients [28], suggesting an interconnected cellular network between tumors and the immune system that may define disease progression. However, on the therapeutic side, there is still limited knowledge regarding the immune signaling events that accompany prostate cancer regression following effective treatment.

When evaluating the expression of a focused panel of immune genes from bulk data, we identified a significant enrichment in antigen presentation pathways, lymphocyte activation, and migration. Among the most upregulated genes, we saw the ligands Cxcl13, Ccl21a, and Ccl22 and the chemokine receptors Cxcr4 and Ccr7. CCL22 is a homeostatic cytokine that can be produced in response to inflammation and acts through its receptor CCR4 to control immune activation in the lymph node [29,30]. The CCL21/CCR7 axis has a dual role, since it can both regulate the homing of immune cells to the lymph node to prime and activate T cells, B cells, and dendritic cells [31] and be involved in a metastatic tumor phenotype by promoting cancer cells migration [32,33]. In parallel to these functions, CXCL13 plays a key role in promoting immune infiltration in the tumor via binding to CXCR5 and controlling tumor behavior by binding to receptors on the tumor surface [34]. While overall pathway analysis suggested that treatment with MCTP39 enriched gene signatures related to immune functions, immune cell recruitment, and antigen processing and presentation, the origin of the signal and the location of the receptor can determine whether the resulting signaling cascade will lead to a pro-tumorigenic or anti-tumor response. Single cell RNASeq analyses revealed a large infiltrating myeloid cell component, consistent with previous studies of CD45^+^ cells infiltrating prostate tumors [10,11], and suggested that improving the function of T cells within prostate cancer will necessitate successfully reprogramming the more abundant myeloid cells to promote local inflammation and anti-tumor immunity [35]. Based on our data and the broad and pleiotropic effects of MCTP39 on myeloid cell reprogramming, we propose that MCTP39 has previously underappreciated activity in targeting and modulating multiple simultaneous attributes of myeloid cell biology, which warrants additional investigation.

To maximize the anti-tumor immune response, the presence and activation of cytotoxic cells is required. Mice that were treated with MCTP39 had a higher frequency of cytotoxic CD8^+^ T cells and NK cells, positive for Granzyme K, Ifn-γ, and Lamp1/Cd107a. The latter plays a key role in forming cytotoxic granules to be released upon antigen recognition, potentially indicating an ongoing anti-tumor response. The expression of Pdcd1/Cd279/Pd-1 was also increased in CD8^+^ T cells, and while elevated PD-1 is a marker for exhausted CD8^+^ T cells, it also defines tumor- and neoantigen-specific T cells. In this case, we also observed a significant reduction in the frequency of CD8^+^ T cells expressing exhaustion markers including Lag3 [36,37,38], Btla [39], Cd244 [40], and Cd160 [41,42], while coexpressing granzymes and IFN-γ, suggesting the presence of activated, rather than exhausted, T cells. Furthermore, MCTP39 did not appear to play a direct role in enhancing CD8^+^ T cells activation, supporting the hypothesis that the observed increase in T cell activation was driven by tumor antigens recognition in addition to immunostimulatory cues in the TME that were driven by MCTP39 treatment. Additionally, it is possible that the effects of MCTP39 on T cells may be unique, in that MCTP39 is able to directly diminish T cell exhaustion, although whether this effect is sustained over time is currently unknown and may necessitate blockade of key inhibitory pathways to produce durable T cell responses. In light of this, further efforts to bolster T cell activity in the tumor following MCTP39 may benefit from either combination therapy using checkpoint blockade or through targeting T cell co-stimulatory pathways. Combination therapies usinganti-PD1/PD-L1 could be particularly beneficial, as MCTP39 appears to enhance anti-tumor immunity in vivo, and WHSC1 knockdown in vitro downregulates PD-L1 expression in PCa cancer cells [8]. Since the expression of CD274/PD-L1 by immune cells was maintained following MCTP39 treatment (with the exception of a modest reduction in M1_Committed macrophages expressing CD274/PD-L1 (Figure S3)), combination with anti-PD-L1 therapy could help to reduce the inhibitory signals arising from tumor-infiltrating immune cells and enhance the duration of anti-tumor immunity. Interestingly, recent studies indicate that NK cells appear to benefit from checkpoint blockade therapy, with NK cell responses having a fundamental role in generating maximal anti-tumor immunity [43]. In our system, the pattern of expression of cytotoxic markers in CD8^+^ T cells and NK cells following treatment indicates that these cell subsets have complementary but different polyfunctional phenotypes, suggesting that a combination of WHSC1 inhibition with checkpoint blockade could optimally activate both cell types, resulting in a potent anti-tumor response. Lastly, because of the increased levels of antigen presentation after MCTP39 treatment [8], we speculate that combination with WHSC1 inhibition could benefit PCa patients who receive vaccines (such as Provenge or Prostvac VF) by preferentially augmenting antigen processing and presentation following vaccination to bolster anti-tumor immunity.

While a high abundance of intratumoral myeloid cells can be associated with an immunosuppressive TME, macrophages and classical monocytes can promote a sustained inflammatory response and favor T cell homing to the tumor. In our model, inhibition of WHSC1 altered the transcriptional programs of M1 and M2 macrophages, upregulating genes in both antigen processing and presentation and leukocytes migration pathways. These results are in line with the increased frequency of cytotoxic immune cells in the treated group and suggest that myeloid cell reprogramming through MCT39 actively enhances the anti-tumor immune response.

Due to the coordinated action of chemokine ligands and receptors, immune cells are able to recruit cytotoxic T and NK cells to the target sites [44,45] or, conversely, they can ameliorate an ongoing inflammation process. We surveyed the ligand–receptor pairs for each classified cell type in an attempt to profile the mechanism(s) by which autocrine and paracrine signaling from cytokines and chemokines can rewire the immune behavior in tumors. For example, Nanostring analysis revealed a higher expression of Cd74. CD74, also named MHC II invariant chain [13], acts as chaperone for class II MHC antigen presentation and collaborates with MHC II to present surface antigens to the immune system [46]. In parallel, it is also known to bind to Cxcr4 in monocytes and T cells [47] and to Cxcr2 to promote leukocytes recruitment [48]. Our data indicate increased signaling in macrophages and DCs that promotes CD8+ T cell recruitment via Cxcr2/Cxcr4. These results are consistent with both higher CD8^+^ T cell infiltration and MHC expression on DCs as measured by flow cytometry, suggesting a potential molecular mechanism by which WHSC1 inhibition alters paracrine signaling in myeloid cells, promoting higher T cell infiltration in the tumor and ultimately establishing an immuno-stimulatory TME. The Ccr5 receptor is uniquely expressed on activated T cells [49,50] and was upregulated in the MCTP39-treated group, in parallel with an upregulation of Ccl5 (a ligand for Ccr5) in DCs, M1, and T_M macrophages. Ccr5 was shown to promote T cell activation in concert with Cxcr4 [50], which can act as receptor for HMGB1 [51], Cxcl12 [52,53], and Cd74 [54]. While computational predictions suggest that complementary pro-inflammatory signals converge to establish a potent anti-tumor immune response, further experimental validation of the changes in cytokine and chemokine levels in each cell type would allow for a more precise interpretation of MCTP39 activity in the TME. The use of multiple prostate cancer models paired with toxicity analyses would further help to expand our current understanding of the safety and clinical applicability of the pharmacological inhibition of WHSC1, thus allowing for these findings to be translated as a clinically actionable treatment approach.

In conclusion, we present a detailed study that addresses the role of WHSC1 in altering anti-tumor cytotoxicity by rewiring the chemokine and cytokine signaling governing the communication between myeloid and lymphoid cells that infiltrate tumors. Due to the vast array of pro-tumorigenic functions shared by WHSC1 across cancer types [1,2,3,4,5,6,7], future studies that extend this approach to other tumor indications will reveal whether WHSC1 has a pan- or multi-cancer immuno-modulatory role. The downstream consequences of these findings have direct therapeutic implications, where cancer patients can be stratified based on WHSC1 expression to identify those that would benefit from WHSC1 inhibition as a complementary approach to immunotherapy.

## 4. Methods

*Mice and sample collection*: Immunocompetent C57BL/6 mice were used to interrogate immunological changes across conditions. Male mice (9–12 weeks old) were obtained from breeder colonies at the Center for Immunotherapy at Roswell Park and injected with 1 × 10^6^ cells TRAMP C-2 cells prepared in 100 μL PBS subcutaneously into the right flank using a 27 G needle. Tumor volume was monitored after every 2–3 days with an electronic caliper and calculated as *V*  =  (*W*^2^ × *L*) / 2, where *V* is tumor volume, *W* is tumor width, and *L* is tumor length. When tumors reached 50–100 mm^3^, mice were randomly divided into two groups and treated with either MCTP-39 (10 mg/kg 5×/week/4weeks, IP) or vehicle control. Mice were euthanized after 4 weeks of treatment. Statistical difference between the two growth curves was calculated via permutation test with 10,000 simulations using the compare GrowthCurves function of the statmod R package [55].

*Cell sorting:* Tumors from treated and control groups were harvested, cut into 1–2 mm pieces, and digested in collagenase (1 mg/mL) (Millipore Sigma, St. Louis, MO, USA) and DNAase 1 (0.5 mg/mL, Roche, Basel, Switzerland) for 45 min at 37 °C with constant shaking. The digested tissues were passed through 40 μm cell strainers gently and centrifugated at 400 *g* for 5 min. The supernatant was discarded, and cell pellets were incubated with 1 mL of ACK lysis buffer (Gibco, Life Technologies, Grand Island, NY, USA) for 5 min at RT. The cells were further washed with FACS buffer 2X and stained with zombie aqua for 15 min at RT, followed by staining with AF700 anti-mouse CD45 antibody (clone 30-F11, Biolegend, San Diego, CA, USA) for 20 min at 4 °C. The cells were further washed 2X with FACS buffer prior to being sorted using a BD FACSAria I cell sorter. The collected cells were gated on live CD45+ fraction and were used for single-cell RNA seq by 10X Genomics (Plesanton, CA, USA).

*Flow cytometry analysis:* For flowcytometry, single-cell suspensions from tumors were prepared as noted above and stained with zombie aqua for 15 min at RT followed by staining with Ax700 anti-CD45 (clone 30-F11), PerCP/Cy5.5 anti-CD8a (clone 53-6.7), FITC anti-CD3 (clone 145-2C11), APC/Fire 750 anti-CD4 (clone GK 1.5), BV605-anti-CD25 (clone PC61), BV605 anti-PD-1 (29F.IA12), BV785 anti-CD11b (clone M1/70), BV605 anti-CD11c (clone N418), PE anti-MHC-II (clone M5/114.15.2) for 20 min at 4 °C. All antibodies were procured from Biolegend, USA, unless mentioned otherwise. After staining, cells were washed and fixed with fixation buffer (Biolegend, San Diego, CA, USA) for 15 min at 4 °C followed by washing 2× with FACS buffer prior to flow cytometry analysis. For in vitro CD8+ T cell proliferation and functional assays, CD8+ T cells were isolated from the spleen and lymph nodes of naive C57BL6/mice using an untouched CD8+ T cell isolation kit (Life Technologies, USA). In total, 2.5 × 10^6^ cells in complete RPMI medium were seeded per well in a 24-well plate and incubated with or without 2 μM MCTP-39. After 4 h of incubation, cells were washed with medium to remove MCTP-39 and were labelled with CFSE for 10 min at RT. The cells were washed to remove uninternalized CFSE and resuspended at 0.1 × 10^6^ cells per well in 100 µL of RPMI medium supplemented with MACSibead particles conjugated to αCD3/CD28 mouse beads (1:1 bead-to-cell ratio, Militinyi Biotech), 0.01M β-mercaptoethanol (Sigma Aldrich, USA), and IL-2 (100 U/mL, Peprotech), and T cells were incubated for 4 days at 37 °C, 5% CO_2_. After incubation, the cells were washed with 1X PBS and stained with zombie aqua followed by anti-CD8 antibody before being analyzed by flow cytometry. For CD8+ T cell functional assays, after 4 days of incubation with MACSibead particles conjugated to αCD3/CD28 mouse beads, the cells were centrifuged, and 100 µL of medium containing a cell activation cocktail with brefeldin A (Biolegend, San Diego, CA, USA) was added; incubation was carried out for 6 h. After incubation, the cells were washed with PBS twice and stained with zombie aqua followed by staining with PerCP anti-CD8 (clone 53-6.7)). For intracellular staining, cells were fixed using fixation buffer (Biolegend, San Diego, CA, USA), permeabilized with intracellular staining perm wash buffer (Biolegend, San Diego, CA, USA), and stained with APC anti-IFN-γ (clone XMG1.2) and PE-anti TNF-α (clone 17B5) for 30 min at RT followed by flow cytometry analysis using a Fortessa flow cytometer. All flow cytometry data were analyzed using FCS express Edition 7 (De Novo Software, Pasadena, CA, USA).

*scRNASeq analysis*: Transcripts from sorted CD45+ cells were quantified using the 10× Genomics scRNASeq Chromium Next GEM Single Cell 3’ Kits v3.1. Raw reads were initially mapped with 10× Cell Ranges prior to importing the results in R for further processing and downstream analyses with Seurat [56]. Briefly, after importing the raw data, cells were filtered based on number of features (200 < *x* < 4000) and percentage of mitochondrial gene expression (*x* < 3). Immune populations were identified using Seurat’s multimodal analysis pipeline followed by manual annotation, and gene expression was imputed using Rmagic [57]. Immune population frequency across treatment groups was calculated using R’s empirical cumulative distribution function (ecdf) on log1p-transformed data, and the Kolmogorov Smirnov’s test was used to test the difference between the two cumulative distributions at a significance level of 0.05.

The list of genes used to classify cell types is provided in Supplementary File S2.

Differential gene expression was performed with Seurat’s FindMarkers function using the MAST algorithm, a minimum pct of 0.25, and a minimum logFC of 0.25. Gene set enrichment analysis was performed using the fgsea package [58] with the ranked top 100 up- and downregulated genes for each cluster. Pathway information for mouse was downloaded from MSigDB and includes gene signatures from GO, Panther, WikiPathways, Inoh, Netpath, and Biocarta.

*Ligand–receptor analysis:* data from ligand–receptor pairs were obtained from Kumar et al. [21]. In our data, ligands were selected from the above based on a cutoff of *p* < 0.05 from differential gene expression analysis. The ligand–receptor score was calculated as the product of the mean expression of the ligand in the selected cell type and the mean expression of the receptor in the target cell line, as described by others [21]. This was done separately for the MCTP39 and control groups. The difference of the two scores was used to infer increased/reduced signaling through a ligand–receptor pair for each cell type combination. Network analysis of the ligand–receptor pairs was done using the igraph package in R [59], and communities were identified via clustering analysis using the cluster_label_prop function in igraph.

*Nanostring data analysis*: Tumors from mice grafted with TRAMP C2 cells were harvested after 4 weeks of treatment with MCTP39. Nucleic acid extraction and sample preparation for the Nanostring myeloid panel array were completed at the Roswell Park Genomics Shared Resources (GSR) following the manufacturer’s guidelines. Raw data were processed in R using the pipeline described by Bhattacharya et al. [60], with downstream differential gene expression analysis performed using DESeq2 [61] at a significance level of *p* < 0.05. Functional enrichment analysis was performed using the DOSE Bioconductor package [62].

## Data Availability

Sequencing data are available on GEO.

## Supplementary Materials

The following are available online at https://www.mdpi.com/article/10.3390/ijms22168742/s1.

## Abbreviations

DC	Dendritic Cells
GSVA	Gene Set Variation Analysis
IFNg	Interferon gamma
MHC	Major Histocompatibility Complex
NK	Natural Killer cells
PCa	Prostate Cancer
QC	Quality Control
scRNASeq	Single-Cell RNA Sequencing
T_M	T cell-like Macrophages
TIL	Tumor-Infiltrating Lymphocypes
TME	Tumor MicroEnvironment
TRAMP	Transgenic Adenocarcinoma of the Mouse Prostate
